# Beyond the base pairs: comparative genome-wide DNA methylation profiling across sequencing technologies

**DOI:** 10.1093/bib/bbae440

**Published:** 2024-09-10

**Authors:** Xin Liu, Yu Pang, Junqi Shan, Yunfei Wang, Yanhua Zheng, Yuhang Xue, Xuerong Zhou, Wenjun Wang, Yanlai Sun, Xiaojing Yan, Jiantao Shi, Xiaoxue Wang, Hongcang Gu, Fan Zhang

**Affiliations:** Anhui Province Key Laboratory of Medical Physics and Technology, Institute of Health and Medical Technology, Hefei Institutes of Physical Science, Chinese Academy of Sciences, Hefei, Anhui Province 230031, China; Hefei Cancer Hospital, Chinese Academy of Sciences, Hefei, Anhui Province 230031, China; Department of Bacteriology and Immunology, Beijing Chest Hospital, Capital Medical University/Beijing Tuberculosis and Thoracic Tumor Research Institute, Beijing 101149, China; Department of Gastrointestinal Surgery, Shandong Cancer Hospital and Institute, Shandong First Medical University and Shandong Academy of Medical Sciences, Jinan, Shandong 250117, China; Hangzhou ShengTing Biotech Co. Ltd, Hangzhou, Zhejiang Province 310018, China; Department of Hematology, The First Hospital of China Medical University, Shenyang, Liaoning, Shenyang, Liaoning province 110001, China; Department of Hematology, The First Hospital of China Medical University, Shenyang, Liaoning, Shenyang, Liaoning province 110001, China; Department of Hematology, The First Hospital of China Medical University, Shenyang, Liaoning, Shenyang, Liaoning province 110001, China; Hangzhou ShengTing Biotech Co. Ltd, Hangzhou, Zhejiang Province 310018, China; Department of Gastrointestinal Surgery, Shandong Cancer Hospital and Institute, Shandong First Medical University and Shandong Academy of Medical Sciences, Jinan, Shandong 250117, China; Department of Hematology, The First Hospital of China Medical University, Shenyang, Liaoning, Shenyang, Liaoning province 110001, China; State Key Laboratory of Molecular Biology, Shanghai Institute of Biochemistry and Cell Biology, Center for Excellence in Molecular Cell Science, Chinese Academy of Sciences, Shanghai 200031, China; Department of Hematology, The First Hospital of China Medical University, Shenyang, Liaoning, Shenyang, Liaoning province 110001, China; Anhui Province Key Laboratory of Medical Physics and Technology, Institute of Health and Medical Technology, Hefei Institutes of Physical Science, Chinese Academy of Sciences, Hefei, Anhui Province 230031, China; Hefei Cancer Hospital, Chinese Academy of Sciences, Hefei, Anhui Province 230031, China; Anhui Province Key Laboratory of Medical Physics and Technology, Institute of Health and Medical Technology, Hefei Institutes of Physical Science, Chinese Academy of Sciences, Hefei, Anhui Province 230031, China; Hefei Cancer Hospital, Chinese Academy of Sciences, Hefei, Anhui Province 230031, China

**Keywords:** sequencing performance, DNB, Illumina, coverage uniformity, methylation

## Abstract

Deoxyribonucleic acid (DNA) methylation plays a key role in gene regulation and is critical for development and human disease. Techniques such as whole-genome bisulfite sequencing (WGBS) and reduced representation bisulfite sequencing (RRBS) allow DNA methylation analysis at the genome scale, with Illumina NovaSeq 6000 and MGI Tech DNBSEQ-T7 being popular due to their efficiency and affordability. However, detailed comparative studies of their performance are not available. In this study, we constructed 60 WGBS and RRBS libraries for two platforms using different types of clinical samples and generated approximately 2.8 terabases of sequencing data. We systematically compared quality control metrics, genomic coverage, CpG methylation levels, intra- and interplatform correlations, and performance in detecting differentially methylated positions. Our results revealed that the DNBSEQ platform exhibited better raw read quality, although base quality recalibration indicated potential overestimation of base quality. The DNBSEQ platform also showed lower sequencing depth and less coverage uniformity in GC-rich regions than did the NovaSeq platform and tended to enrich methylated regions. Overall, both platforms demonstrated robust intra- and interplatform reproducibility for RRBS and WGBS, with NovaSeq performing better for WGBS, highlighting the importance of considering these factors when selecting a platform for bisulfite sequencing.

## Introduction

Deoxyribonucleic acid (DNA) methylation, an epigenetic modification involving the addition of a methyl group to cytosines in DNA molecules, plays a pivotal role in the regulation of gene expression, genome stability, and various cellular processes [1–3]. Comprehensive profiling of DNA methylation patterns has become essential for understanding the molecular mechanisms underlying various biological phenomena, including development and human diseases, particularly cancer [4–7]. The emergence of next-generation sequencing (NGS) technologies has revolutionized our ability to investigate genome-wide DNA methylation and dramatically reduced sequencing costs [8, 9]. NGS sequencers manufactured by Illumina, such as the GAII, HiSeq 2500, HiSeq 3000, HiSeq 4000, and HiSeq X10, are widely used in the life sciences and have generated the majority of published sequencing data since 2006 [10, 11]. MGI Tech sequencers, including the BGISEQ-500 and MGISEQ-2000, have demonstrated comparable sequencing performance [12, 13]. Of note, the recent launch of two commercial sequencing instruments, the Illumina NovaSeq 6000 in 2017 and the MGI DNBSEQ-T7 in 2019, represent remarkable advances in sequencing capacity and cost per base [14].

The NovaSeq 6000, developed by Illumina, a leader in NGS sequencer manufacturing, can produce up to 6 terabases (Tb) of data per run, costing approximately $10 per gigabase (Gb) [15]. Similarly, DNBSEQ-T7 from MGI Tech, another key player in the field, offers comparable data output at a lower sequencing cost [13, 15]. However, these platforms differ significantly in their sequencing principles. Illumina sequencing employs sequencing-by-synthesis (SBS) with reversible dye terminators for base identification. This involves bridge amplification on a flow cell surface, leading to the exponential amplification of clonal sequencing templates and the formation of dense DNA clusters [16]. In contrast, MGI Tech’s DNBSEQ-T7, along with its BGI-500 and MGISEQ-2000 sequencers, uses DNA nanoball (DNB) amplification and combinatorial probe anchor synthesis (cPAS) for high-throughput sequencing [10, 17, 18]. Notably, the MGI Tech sequencers rely on rolling circle amplification (RCA) to generate DNBs for linear amplification, potentially leading to lower error rates [10]. Moreover, while the high-throughput NovaSeq 6000 utilizes a two-color fluorescence system for image scanning, simplifying optical detection, DNBSEQ-T7 employs a four-color system, with each of the four bases labeled with a unique fluorescent dye [19, 20].

A few studies have been conducted between the two platforms for whole-genome sequencing (WGS) and whole exome sequencing (WES) in various sequencing applications, including WGS, WES [14, 15, 21], single-cell and bulk RNA sequencing [22–25], and microbiome analysis [26]. However, no study has evaluated these platforms for genome-wide DNA methylation analysis using bisulfite sequencing (BS-seq), especially for two widely used strategies: reduced representation bisulfite sequencing (RRBS) [8, 27] and whole-genome bisulfite sequencing (WGBS) [9]. RRBS employs restriction enzymes to digest genomic DNA and size-select CpG-rich regions [27]. It is a cost-effective approach and has been widely used for methylation studies in large cohorts [28–31]. WGBS, on the other hand, covers almost all cytosines in the genome, making it the gold standard for comprehensive DNA methylation profiling [9, 32]. Notably, factors such as the bisulfite conversion rate and PCR and library construction strategies can introduce bias in both RRBS and WGBS [33–37].

In this study, we evaluated the performance of the NovaSeq 6000 and DNBSEQ-T7 sequencers in RRBS and WGBS and their impact on DNA methylation analysis, focusing on myelodysplastic syndrome (MDS), a disease that has been associated with methylation abnormalities [38, 39]. We collected bone marrow mononuclear cells (BMMNCs), white blood cells (WBCs), and plasma cell-free DNA (cfDNA) from MDS patients and healthy donors and constructed WGBS and RRBS libraries with different DNA inputs specifically for each of the two sequencing platforms. Our evaluation included sequencing quality, regional CpG coverage, coverage uniformity, and detection of differentially methylated CpG sites (DMPs). Our analysis highlights the key factors that should be considered when selecting a platform for BS-seq.

## Materials and methods

### Patients and healthy donors

The study was approved by the Ethics Committee of the First Hospital of China Medical University (Shenyang, China) and was conducted in accordance with the tenets of the Declaration of Helsinki for biomedical research. All participants provided written informed consent. Bone marrow (BM) aspirates and peripheral blood (PB) samples were collected from five patients with newly diagnosed MDS. BM aspirates and PB samples were also collected from two healthy BM donors for comparison.

### Peripheral blood and bone marrow processing and Deoxyribonucleic acid isolation

BM aspirates were collected, and genomic DNA was extracted from 200 μl of BM aspirates using the TIANamp Genomic DNA Kit (TIANGEN, Beijing, China) according to the manufacturer’s instructions. The genomic DNA was eluted in 50 μl of TE buffer.

PB samples were collected in K3EDTA tubes and processed within 24 hours to isolate plasma and WBCs. Briefly, blood samples were centrifuged at 1600 × g for 10 minutes at 4°C. The plasma layer was then transferred to Eppendorf tubes for further processing to isolate cfDNA. The buffy coat was also pipetted into a fresh Eppendorf tube for genomic DNA isolation using the TIANamp Genomic DNA Kit (TIANGEN, Beijing, China). Genomic DNA was then eluted in 50 μl of TE buffer. Plasma samples were further centrifuged at 16 000 × g for 10 minutes and used for cfDNA purification using the Magbead Free-Circulating DNA Maxi Kit (Jiangsu CoWin Biotech., Nanjing, China) according to the manufacturer’s instructions. The final cfDNA was eluted in 50 μl of RNase-free water. The concentrations of genomic DNA and cfDNA were quantified using the Qubit dsDNA HS (High Sensitivity) Assay Kit (Thermo Fisher Scientific, Waltham, MA, USA). Both purified cfDNA and genomic DNA were stored at −20°C if not used immediately.

### Library preparation and sequencing

We prepared WGBS libraries for Illumina sequencing using 5–100 ng of purified genomic DNA or cfDNA, which was bisulfite converted using the EpiTect Fast Bisulfite Conversion Kit and a protocol described in a previous study [27]. The bisulfite-converted DNA was eluted in 40 μL of EB buffer and used for WGBS library construction using the Methyl-Seq DNA Library Kit (Swift Biosciences, Ann Arbor, MI, USA) according to the manufacturer’s guidelines. To generate RRBS libraries for Illumina sequencing, 2–50 ng of genomic DNA or cfDNA was utilized according to a protocol outlined in an earlier publication [40]. Approximately 30% of the PhiX library (PhiX Control V3, Illumina, San Diego, CA, USA) or nonbisulfite sequencing library DNA was spiked into both the WGBS and RRBS libraries to adjust for base composition bias. The pooled libraries were sequenced using a 150 bp paired-end protocol on the NovaSeq 6000 system according to Illumina specifications.

The WGBS and RRBS libraries for DNBSEQ-T7 sequencing were derived from the Illumina libraries mentioned above. Specifically, 50 ng of Illumina library DNA was processed using 5 cycles of PCR to incorporate MGI adapters. The amplified products were then circularized to generate single-stranded DNA libraries using the MGIEasy Circularization Kit (Cat# 1000004155, MGI Tech., Beijing China) according to the manufacturer’s instructions. To counteract the reduced base diversity resulting from sodium bisulfite conversion, 30% of the PhiX or nonbisulfite sequencing library DNA was added to the DNBSEQ WGBS and RRBS libraries. The library pool was then subjected to 150 bp paired-end sequencing on the DNBSEQ-T7 platform.

### Whole-genome bisulfite sequencing data alignment and CpG calling

WGBS raw data from NovaSeq-6000 and DNBSEQ-T7 were preprocessed, aligned to the human reference genome, and converted to CpG methylation count matrices using the default parameters in CpG_Me (https://github.com/ben-laufer/CpG_Me, v1.4.1). Reads were trimmed to remove adapters and methylation bias at both the 5′ and 3′ ends. After trimming, the reads were aligned to the human reference genome hg19 and filtered for PCR duplicates. Cytosine methylation reports were generated using all covered CpG sites. The CpG_Me workflow incorporates Trim Galore (v0.6.10), Bismark (v0.24.0), Bowtie2 (v2.4.5), SAMtools (v1.6), and MultiQC (v1.14) [41–44]. RRBS raw data were processed using a homemade pipeline. Reads were trimmed to remove adapters at both the 5′ and 3′ ends using trim_galore with the parameter ‘—rrbs’. FastQC was used before and after trimming to check the quality of the reads. Clean reads were mapped to the human reference genome hg19 using BSmap (v2.90) with the parameters ‘-q 20 -f 5 -r 0 -v 0.05 -s 16 -S 1 -n 0’ [45]. CpG calling was implemented by MethylDackel (https://github.com/dpryan79/methyldackel, v0.6.1) with default parameters.

The cytosine methylation matrices of WGBS and RRBS were transformed into the format recognized by methylKit [46]. The pairwise correlation coefficient was calculated using methylKit with a cutoff of coverage>10.

### Calculation of quality control metrics

For base quality statistics, Seqkit was used to count the percentages of reads with Q20 and Q30 quality scores [47]. The percentage of trimming due to quality (Phred score quality cutoff <20) and the mapping ratio of unique paired reads were extracted from the mapping outputs. The percentage of non-CpG methylation was extracted from bismarks for WGBS and BSmap for RRBS. The average insert size was determined using Qualimap (v2.3). The mapping error rate was extracted from the mappers.

### Recalibration of the whole-genome bisulfite sequencing base quality score

To perform base quality score recalibration (BQSR) for WGBS, we first added an MD tag to the bam file using SAMtools calmd [44], which records mismatch information. The WGBS bam file was then preprocessed using the ‘double-mask’ method provided by the revelio.py script [48]. In the first step, specific nucleotides in bisulfite contexts are replaced by the corresponding reference base, and in the second step, any given nucleotide that may have arisen due to bisulfite conversion is assigned a BQ score of 0. We then added an RG tag to the bam file using Picard (v3.0.0) AddOrReplace-ReadGroups (—RGID sample1 —RGLB lib1 —RGPL illumina —RGPU unit1 —RGSM sample1 —VALIDATION_STRINGENCY LENIENT) [49]. The gatk BaseRecalibrator and gatk ApplyBQSR (v4.4.0.0) were used to perform the BQSR step [49]. For the gatk BaseRecalibrator, dbSNP version 151 for hg19 was used to filter SNPs. Finally, gatk AnalyzeCovariates was used to visualize the report files.

### Mean coverage and mean methylation visualization

To plot the mean coverage of WGBS and RRBS, the bigwig for the coverage signal was generated using the bamCoverage tools from deepTools (v3.5.2) [50]. The coverage signals were plotted as heatmaps using the deepTools plotHeatmap. The average summary plots for the coverage signals were plotted using the plotProfile function in deepTools. The matrix used for these functions was generated using the deepTools computeMatrix function. To plot the mean methylation of WGBS and RRBS, the bigwig for the methylation signal was generated using the bamCoverage tools from deepTools. Then, plotHeatmap, plotProfile and computeMatrix functions in deepTools were used as described above. The genomic elements, including transcription start sites (TSSs), CpG islands (CGIs), and enhancers, were downloaded from the UCSC browser. Promoters were defined as those whose TSS was upstream or downstream of 1 kb. The CpG shore, shelf and inter-CGI were defined as before.

### Identification of differentially methylated CpG sites between myelodysplastic syndrome patients and healthy donors

To compare the performance for detecting differentially methylated positions between two sequencing platforms, the calculate DiffMeth() function in the R package methylKit (v1.28.0) was used to calculate DMPs (delta beta greater than 0.2 were preserved) from BM aspirates of 3 MDS patients and 2 healthy donors. Another cohort with HM450K data from BM samples of 156 MDS patients and 10 healthy donors was used as a reference for the DMPs. The methylation matrix was downloaded from NCBI (GSE152710) and processed using the ChAMP (v2.34.0) package in the R environment. The probes in the HM450k array data were filtered out if P > 0.01 was detected. The data were then normalized using PBC methods. DMPs were identified using Champ. DMP() function in CHAMP with default parameters. Scatterplots of DMPs between two sequencing platforms or between one sequencing platform and the reference BeadChip were generated using baseR (4.3.2). The distribution of genomic features for DMPs was annotated using ChIPseeker (v1.38.0) as described above. The identified DMPs in the two sequencing platforms were mapped to the corresponding gene promoters by chromosomal location if they were in the TSS ± 1 kb region.

## Results

### Sample information and sequencing

**Figure 1:**
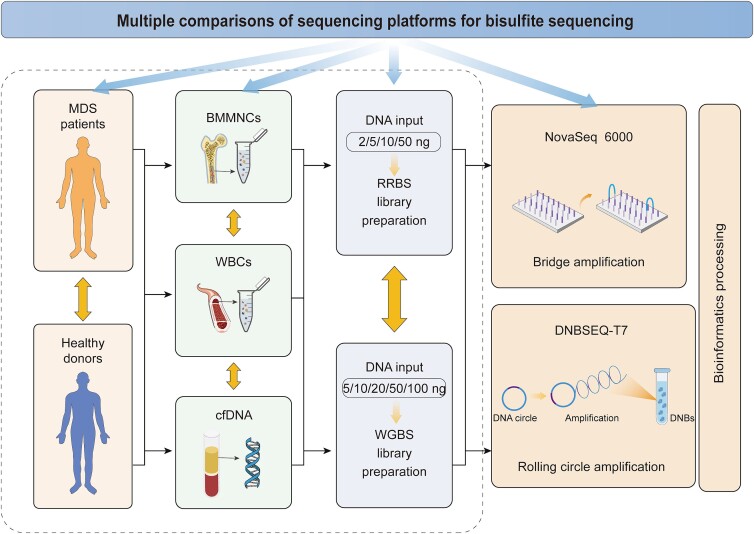
Comparison of the NovaSeq 6000 and DNBSEQ-T7 platform study designs for bisulfite sequencing.

To study how different sequencing platforms affect genome-wide methylation analysis, we collected BM from five MDS patients and two healthy donors, along with their PB. We extracted genomic DNA from BMMNCs or WBCs and cfDNA from plasma. We then generated WGBS libraries with varying amounts of DNA from BMMNCs (5, 50, 100 ng), WBCs (5, 50 ng) and cfDNA (5, 10, 20 ng) and RRBS libraries with BMMNCs (2, 10, 50 ng), WBCs (2, 10 ng) and cfDNA (2, 5, 10 ng). Notably, for a direct head-to-head comparison, both the WGBS and RRBS libraries for DNBSEQ-T7 sequencing were converted from aliquots of the corresponding libraries originally generated for the NovaSeq 6000 platform (Fig. 1 and Table 1). In total, we sequenced 60 libraries and obtained approximately 2.8 Tb of pass filter data. This dataset included 16 WGBS libraries with an average of 117.8 ± 17.7 Gb per library, 36 RRBS libraries with an average of 10.7 ± 3.3 Gb each, and 8 RRBS libraries intended for deep sequencing with an average of 71.7 ± 17.4 Gb per library. To ensure comparability, we randomly selected 30 million read pairs per library for downstream bioinformatics analysis of the RRBS data, while 200 million read pairs per library were used for analysis of the WGBS data. In addition, to explore the effect of sequencing depth, we performed downsampling analysis on the eight deeply sequenced RRBS libraries, reducing the read count from 220 million to 30 million. (Fig. 1 and Table 1).

**Table 1:** Characteristics of the RRBS and WGBS libraries used in this study

Method	Individuals	Sample type	Input (ng)	Reads
RRBS	M_167	CF; BM; WB	10; 10; 10	30 M
	M_391	CF; BM; WB	2\10; 2\10\50; 10	30 M
	M_408	CF; BM; WB	5; 10; 2\10	30 M
	M_351	CF; WB	10; 10	30\60\90\120\220 M
	M_915	CF; WB	10; 10	30\60\90\120\220 M
	H_054	CF; BM; WB	10; 10; 10	30 M
	H_833	BM; WB	10; 10	30 M
WGBS	M_408	CF; BM; WB	5\20; 5\50\100; 5\50	200 M
	H_833	CF	10	200 M

CF: plasma cfDNA; BM: genomic DNA of BMMNCs; WB: genomic DNA of WBCs.

### Sequencing performance of reduced representation bisulfite sequencing and whole-genome bisulfite sequencing

**Figure 2:**
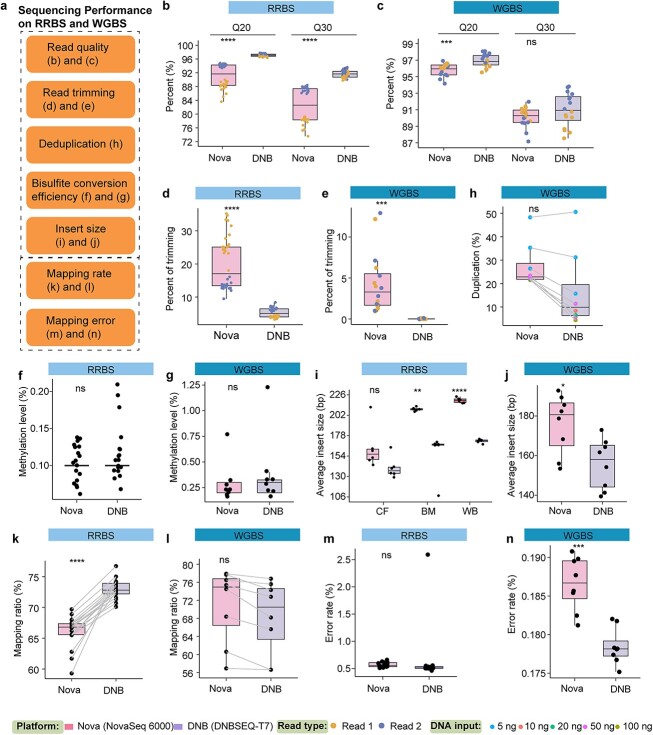
Comparison of sequencing metrics for RRBS and WGBS data generated by the NovaSeq 6000 and DNBSEQ-T7 platforms. (a) Illustrates critical metrics for evaluating sequencing performance on RRBS and WGBS datasets. The box plots in (b) and (c) show the percentages of Q20 and Q30 reads for all RRBS and WGBS datasets, respectively. (d) and (e) Box plots illustrating the percentage of reads trimmed for Phred scores <20 on the RRBS and WGBS datasets, respectively. (f) and (g) Box plots showing the percentage of methylated cytosines in the non-CpG contexts for the RRBS and WGBS datasets, respectively. (h) Box plot of the duplication rate for the WGBS datasets, with different DNA inputs marked by different colors. (i) Box plots representing the average insert size for cfDNA, BM, and WBCs RRBS samples, and (j) box plots of the average insert size for all WGBS samples. (k) and (l) Box plots of the uniquely mapped read ratio, while (m) and (n) box plots of the mapping error ratio for the RRBS and WGBS datasets, respectively.

The sequencing performance of the NovaSeq 6000 and DNBSEQ-T7 platforms on RRBS and WGBS libraries was evaluated based on several metrics: raw read quality, read trimming efficiency, bisulfite conversion ratio (BCR), deduplication rate, insert size, mapping efficiency, and sequencing error rate. (Fig. 2a).

#### Raw read quality

To assess the quality of the raw sequencing reads generated by the NovaSeq 6000 and DNBSEQ-T7 platforms, we utilized the software ‘SeqKit’ (v2.6.1) to evaluate the base quality of RRBS and WGBS reads based on the Q20 and Q30 metrics [47]. Our results showed that the DNBseq-T7 platform demonstrated significantly greater average rates of quality scores above Q20 and Q30 for both RRBS (97% and 92%) and WGBS (96% and 91%) samples than did NovaSeq 6000 (92% and 83% for RRBS, 95% and 90% for WGBS) (Fig. 2b and c). We also noticed that the quality scores of Read 1 sequences in the RRBS data were much lower than those of Read 2 sequences for the NovaSeq 6000 (Q20: mean 88% versus 94%; Q30: mean 78% versus 87%), whereas the DNBSEQ-T7 platform showed minimal differences between the two (Fig. 2b and c). Furthermore, to address systematic errors and biases in the base calling from the sequencers, we recalibrated the reported base quality scores in the WGBS data using the script revelio.py [48] and the GATK BQSR function [51]. This involved converting specific nucleotides in bisulfite contexts to their corresponding reference bases and adjusting the base quality scores (refer to the Materials and methods section for more details). The results indicated that both platforms overestimated the percentage of high-quality bases (Supplementary Fig. S1a and b), with the DNBSEQ-T7 platform exhibiting lower accuracy in quality score calling than the NovaSeq 6000 platform (Supplementary Fig. S1c). The mean quality scores before and after BQSR were similar for each cycle (Supplementary Fig. S1d). When comparing the base quality scores before and after recalibration in different base contexts, we observed that the DNBSEQ-T7 platform generally had lower quality score accuracy than did the NovaSeq 6000 platform, and the difference in quality scores before and after recalibration was more pronounced for the DNBSEQ-T7 platform (Supplementary Fig. S1e). However, when considering the mean quality score, the difference in scores between the two platforms before and after recalibration was not significant (Supplementary Fig. S1f).

#### Read trimming

Trimming low-quality bases from sequencing reads is a routine procedure. In this study, we used trim_galore [52] to eliminate low-quality ends, which we defined as those with a Phred score below 20. The percentage of trimmed reads varied between the two platforms. For RRBS data on the DNBSEQ platform, the trimming percentage ranged from 3% to 7%, while for WGBS data, it was only 1% (Fig. 2d and e). On the NovaSeq platform, RRBS data showed a higher trimming percentage ranging from 10% to 35%, while for WGBS data, the percentage ranged from 1% to 6% (Fig. 2d and e). Notably, the trimming percentages of the RRBS data from the NovaSeq platform differed between Read 1 and Read 2 (Fig. 2d and e).

#### Bisulfite conversion ratio

Assessing the BCR is crucial for the RRBS and WGBS datasets because a lower BCR tends to overestimate methylation levels [53]. Here, we determined the efficiency of bisulfite conversion by analyzing the observed BCR at non-CpG sites [54]. Our findings indicate that non-converted cytosines in non-CpG contexts are rare (<1%) in most RRBS samples, with no significant difference observed between the DNBSEQ and NovaSeq platforms (Fig. 2f and g). These results suggest highly efficient bisulfite conversion (> 99.8%) for RRBS. However, for WGBS experiments, the bisulfite conversion efficiency was slightly lower (>98.8%) for both platforms.

#### Whole-genome bisulfite sequencing duplication rate

The nature of RRBS library preparation makes it difficult to distinguish whether two identical reads originate from different molecules or are the result of PCR duplication, although a lower number of PCRs is usually associated with more tolerable duplication rates [27, 40, 55]. However, it is possible for WGBS to eliminate duplicate reads by analyzing terminal sequences prior to methylation calling. We compared the duplication rates between the DNBSEQ and NovaSeq platforms and found that the DNBSEQ platform had a lower duplication rate (Fig. 2h). As anticipated, samples with lower DNA input (5 ng) had to undergo more PCR cycles and consequently showed a greater duplication rate (Fig. 2h).

#### Insert size

We conducted an analysis of the insert fragment lengths from various library types (cfDNA, genomic DNA of BMMNCs or WBCs) using the bam data to investigate whether different sequencing platforms exhibit a bias toward specific fragment lengths [56]. Our results revealed that for RRBS, the fragment lengths of cfDNA were consistently shorter than those of genomic DNA (Fig. 2i), likely because cfDNA contains shorter fragments than genomic DNA, which is consistent with previous observations [57]. Additionally, we observed that the NovaSeq platform generally produced longer insert sizes in RRBS libraries for both plasma cfDNA and genomic DNA, a trend that was also evident in WGBS libraries (Fig. 2i and j).

#### Mapping quality

Following the trimming process, the trimmed RRBS and WGBS reads were mapped separately to the human reference genome (hg19) using the Bsmap and CpG_Me alignment pipelines [58]. The resulting bam files were then sorted using SAMtools. Subsequently, MultiQC [43] was utilized to assess the mapping quality. Our analysis revealed a notably greater percentage of unique mapped read pairs in the RRBS data obtained from the DNBSEQ platform than in those obtained from the NovaSeq platform, with an average of 72% for DNBSEQ and 66% for NovaSeq (Fig. 2k). However, for the WGBS data, there was no significant difference in the percentage of mapped reads between the two platforms, with 68% for DNBSEQ and 71% for NovaSeq (Fig. 2l). The error rate of mapping was determined from the alignment tools, and we observed an equally low error rate for RRBS on both platforms but a slightly higher error rate for WGBS on the NovaSeq platform (Fig. 2m and n).

### Read coverage and methylation levels for reduced representation bisulfite sequencing and whole-genome bisulfite sequencing

The coverage of CpG sites plays a crucial role in assessing the quality of sequencing data, as higher coverage typically leads to more accurate estimates of methylation status. Given the substantial variation in quality control metrics between the two sequencing platforms, it is unclear whether this discrepancy impacts the calling of CGIs, coverage of different genomic regions, and quantification of methylation levels. To address these concerns, we conducted a comparison using RRBS data after downsampling to 30 million mapped reads [44].

#### Genome coverage of reduced representation bisulfite sequencing

**Figure 3:**
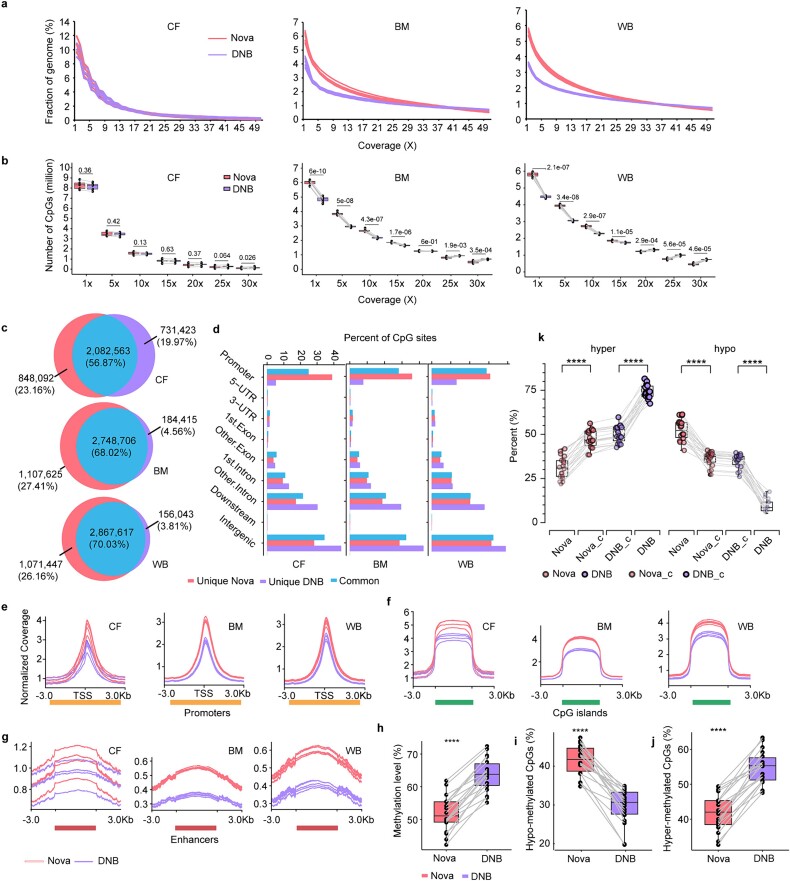
Characteristics of coverage and methylation bias for RRBS between NovaSeq 6000 and DNBSEQ-T7. To systematically explore the coverage bias of the two sequencing platforms when performing RRBS, the raw data were trimmed and mapped to the reference genome (hg19) and downsampled to 30 million reads for all RRBS libraries. (a) the fraction of the reference genome covered by reads for each library was calculated and plotted at minimum coverage depths of 1× to 50×. (b) The number of CpG sites covered for each library was calculated and graphed at minimum coverage depths of 1× to 30×. The paired *t*-test *P* values are shown at the top of the box. (c) The number of CpGs (minimum coverage depths of 10×) for 18 RRBS libraries from each platform were combined, and the Venn diagrams show the number of CpGs covered by each sequencing platform. (d) The genomic distribution of platform-shared and platform-specific CpG sites. The mean normalized coverages across (e) promoters, (f) CGIs, (g) and enhancers were plotted using deepTools [50]. The methylation levels in CpG contexts were plotted for each library (h). (i) and (j) Box plots showing the percentage of hypomethylated CpGs (beta value <0.2) and hypermethylated CpGs (beta value >0.8). (k) Box plots showing the percentage of hypomethylated and hypermethylated CpGs for platform-specific (Nova versus DNB) and platform-shared (Nova_c versus DNB_c) CpGs.

Our analysis revealed that the assembled RRBS reads of cfDNA covered approximately 10%–12% of the hg19 sequence at a minimum of 1x sequencing depth on both the NovaSeq and DNBSEQ platforms (Fig. 3a). In contrast, the assembled RRBS reads of genomic DNA from BMMNC and WBC samples covered a lower percentage of the reference genome at ≥1x coverage, approximately 6% on the NovaSeq platform and even lower (approximately 4%) on the DNBSEQ platform (Fig. 3a).

#### CpG site coverage of reduced representation bisulfite sequencing

RRBS enriched genomic regions containing CpG dinucleotides, capturing the majority, although not all, of CGIs and promoters [27]. We observed significant differences in CpG site coverage for genomic DNA (BMMNC and WBC) RRBS data between the two platforms, while CpG coverage for cfDNA RRBS data was similar (Fig. 3b). On the NovaSeq platform, RRBS data for genomic DNA samples achieved at least 1x coverage for approximately 6 million CpGs across the genome, of which 2.60–2.86 million (42–50%) were covered at depths ≥10×. However, on the DNBSEQ platform, fewer than 5 million CpGs were covered at ≥1×, with 2.11–2.33 million sites (42–52%) covered at ≥10× (Fig. 3b). Notably, the NovaSeq platform detected approximately 500 000 more CpGs with a minimum coverage depth of 10x than did the DNBSEQ platform.

### Reproducibility and genomic distribution of CpG sites in reduced representation bisulfite sequencing

We examined the reproducibility of CpG sites (coverage depth ≥ 10×) across different sample types (cfDNA, genomic DNA of BMMNCs and WBCs) and sequencing platforms. The results indicated comparable numbers of shared CpGs between the NovaSeq and DNBSEQ platforms, with approximately 67% of the CpG sites shared by at least two cfDNA samples for both platforms (Supplementary Fig. S2a) and 85%–90% of the CpGs shared by at least two genomic DNA samples for both platforms (Supplementary Fig. S2b and c). Additionally, when aggregating CpG sites identified in all samples, we found that 57%, 68%, and 70% of the identified CpGs in the cfDNA, BMMNC, and WBC genomic DNA RRBS datasets, respectively, were common between the NovaSeq and DNBSEQ platforms, with a greater proportion of platform-specific CpGs observed on the NovaSeq platform for the BMMNC and WBC genomic DNA RRBS datasets (Fig. 3c). Furthermore, we categorized platform-shared and platform-specific CpG sites into different genomic regions and discovered distinct regional differences. The majority of CpG sites were located in the promoter, intron, and intergenic regions, with NovaSeq-specific CpGs concentrated in the promoter region and DNBSEQ-specific CpGs biased toward the intron and intergenic regions (Fig. 3d).

### Read coverage and methylation level of reduced representation bisulfite sequencing

In addition, we conducted a comparison to evaluate any differences in the average read coverage and mean methylation level between the two sequencing platforms across three genomic regions: CGI, promoter, and enhancer regions. The promoter region is defined as the region spanning 2000 bp upstream and downstream of the TSS. The enhancer regions were determined based on the CAGE-based enhancers for hg19 from the FANTOM5 consortium [59]. We used deepTools [60] to calculate the read coverage over the promoter, CGI and enhancer regions (referred to as scale regions) by scaling each region to the same size. The results showed that the RRBS data obtained from the NovaSeq platform exhibited greater coverage in all three types of regions than did those obtained from the DNBSEQ platform (Fig. 3e–g), leading to a greater number of CpGs being covered (Fig. 3c). The extent of read coverage at genomic sites is likely to influence the estimation of methylation status. The significantly higher read coverage of the NovaSeq platform resulted in a lower methylation level (Fig. 3h). However, the discrepancy in methylation levels was not as pronounced as the difference in read coverage (Supplementary Fig. S3a–c). This trend remained consistent across all three sample types (cfDNA, BMMNCs, and WBCs).

We observed that the DNBSEQ platform exhibited higher levels of methylation in the CpG context than did the NovaSeq platform. The cytosine methylation ratios on the DNBSEQ platform ranged from 0.55 to 0.72, while on the NovaSeq platform, they ranged from 0.42 to 0.62 (paired *t*-test, *P* value<.0001; Fig. 3h). Consistent with this, the DNBSEQ platform displayed a significantly greater proportion of hypermethylated CpGs (55% versus 42%, *P* value <.0001) and a significantly lower proportion of hypomethylated CpGs (30% versus 41%, *P* value <.0001) than did the NovaSeq platform (Fig. 3i and j). The beta value is often used to quantify DNA methylation levels as the ratio of methylated CpG intensity to the combined intensity of methylated and unmethylated CpGs. CpGs with beta values >0.8 are considered hypermethylated, while those with beta values <0.2 are considered hypomethylated [61]. Additionally, when examining the proportions of hyper or hypomethylated sites for platform-common and platform-specific covered CpG sites, we found that the proportions were similar for the shared sites of the two platforms. However, for platform-specific CpGs, DNBSEQ has a higher percentage of hypermethylated CpGs and a lower percentage of hypomethylated CpGs compared to NovaSeq, resulting in an increasing trend in hypermethylated CpGs and a decreasing trend in hypomethylated CpGs across NovaSeq-specific, shared, and DNBSEQ-specific CpGs (Fig. 3k). These results suggest that the overall higher methylation levels in the CpG contexts on the DNBSEQ platform are mainly attributed to platform-specific CpG sites.

### Read coverage and methylation level of whole-genome bisulfite sequencing

**Figure 4:**
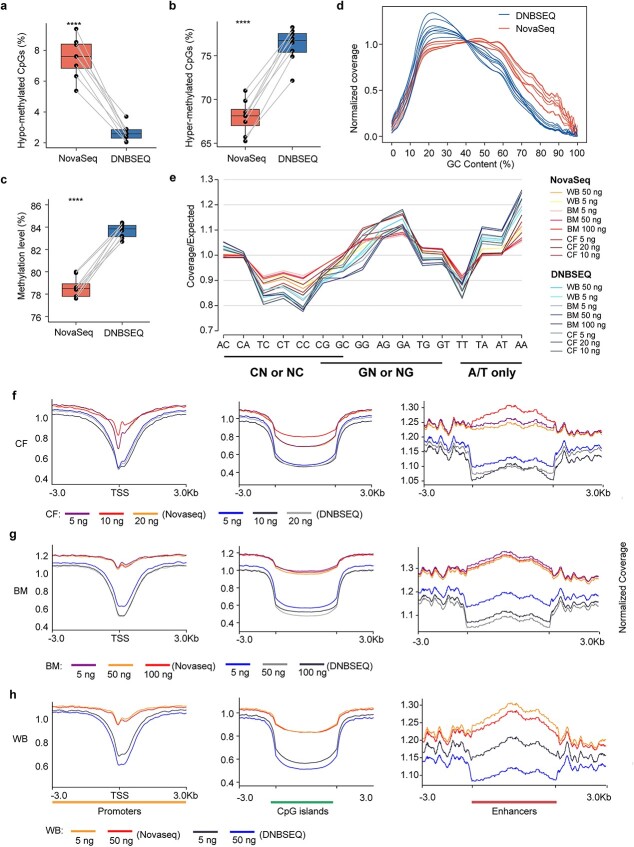
Characteristics of coverage and methylation bias for WGBS between NovaSeq 6000 and DNBSEQ-T7. Eight WGBS libraries were initially prepared for NovaSeq, with aliquots converted for DNBSEQ sequencing, and both sets were then sequenced on their respective platforms. The raw data were trimmed and mapped to the reference genome (hg19) and downsampled to 200 million reads for each library. The percentages of (a) hypermethylated CpGs and (b) hypomethylated CpGs, (c) methylation levels, and (d) GC bias plots were plotted for all 16 WGBS libraries generated with different sample types (CF, BM, and WB) and input levels (5, 10, 20, 50, and 100 ng) and sequenced by DNBSEQ-T7 and NovaSeq 6000. (e) Coverage of dinucleotides in all 16 WGBS datasets, with coverage/expected denoted as a fold difference from the genomic expected [41]. Dinucleotides are underlined as derived from C, G, or A/T only. The normalized coverage of (f) promoters, (g) CGIs, and (h) enhancers was calculated and visualized using deepTools.

Compared to RRBS, WGBS covers a much larger number of CpGs across the entire genome. Although the biases introduced by WGBS library preparation strategies in DNA methylation data have been evaluated [62], a systematic assessment of the biases introduced by the NovaSeq and DNBSEQ sequencing platforms is still lacking. Our analysis revealed that both the NovaSeq and DNBSEQ platforms were able to capture 85% to 90% of the CpG sites in the human genome at 1× coverage (Supplementary Fig. S4). Similar to the RRBS data, the DNBSEQ platform showed a significantly greater percentage of cytosine methylation (84% versus 79%, *P* < .0001), a significantly greater percentage of hypermethylated CpGs (76% versus 68%, *P*< .0001), and a lower percentage of hypomethylated CpGs (2.6% versus 7.5%, *P* < .0001) than did the NovaSeq platform (Fig. 4a–c). However, WGBS exhibited a bias toward detecting more hypermethylated CpGs than hypomethylated CpGs (68%–76% versus 2.6%–7.5%) in comparison to the RRBS data (42%–55% versus 30%–41%) (Fig. 3i and j and Fig. 4a–c). In addition, we used Picard CollectGcBiasMetrics [49] to assess the normalized coverage of the reads at different bins of the reference sequence, which had varying percentages of G + C content ranging from 0% to 100%. The results showed that the NovaSeq platform displayed a more uniform distribution across a wide range of GC contents (GC%: 20%–70%) than did the DNBSEQ platform, which had greater coverage in GC-poor regions (GC%: 20%–40%; Fig. 4d). The dinucleotide plot further supported the bias of the DNBSEQ platform toward AT-rich and GC-poor regions compared to the NovaSeq platform (Fig. 4e).

Similar to the analysis of RRBS data, we performed a comparison of average read coverage and methylation levels in WGBS datasets between the two platforms for CGI, promoter, and enhancer regions. Our results revealed that the NovaSeq platform consistently demonstrated significantly greater read coverage and lower methylation levels across all regions (promoter, CGI, and enhancer) than did the DNBSEQ platform at all input levels (Fig. 4f–h and Supplementary Fig. S5a–d). In addition, the NovaSeq platform produced similar coverage and methylation levels for different input levels of genomic DNA but lower coverage for lower input levels of cfDNA, whereas the DNBSEQ platform exhibited variable coverage and methylation levels for different input levels of genomic DNA (Fig. 4f–h and Supplementary Fig. S5b–d).

We also investigated whether the GC content had any effect on the methylation levels of these regions. We sorted the CGIs according to their GC content and selected the top 1000 CGIs with the highest GC% and the bottom 1000 CGIs with the lowest GC%. We then compared the average methylation level of these two sets with that of all CGIs. Our analysis revealed that the group with the highest GC content had a lower average methylation level, whereas the group with the lowest GC content had a greater average methylation level than the total CGI group (Supplementary Fig. S5d). These results suggest that the differences in read coverage and methylation levels at different regions between the two platforms may be attributed to differences in GC content composition.

### Intraplatform reproducibility of reduced representation bisulfite sequencing and whole-genome bisulfite sequencing

Ensuring robust intraplatform reproducibility is essential for reliable data output from sequencing platforms. While Illumina sequencing platforms have demonstrated good reproducibility in WGS and RNA sequencing [63–65], there is limited research on the performance of the NovaSeq and DNBSEQ platforms in WGBS and RRBS. It is also unclear whether these two sequencing platforms are comparable in detecting DNA methylation patterns. In this study, we compared the intraplatform reproducibility and interplatform correlation between RRBS and WGBS datasets from two sequencing platforms.

#### Intraplatform reproducibility of reduced representation bisulfite sequencing

**Figure 5:**
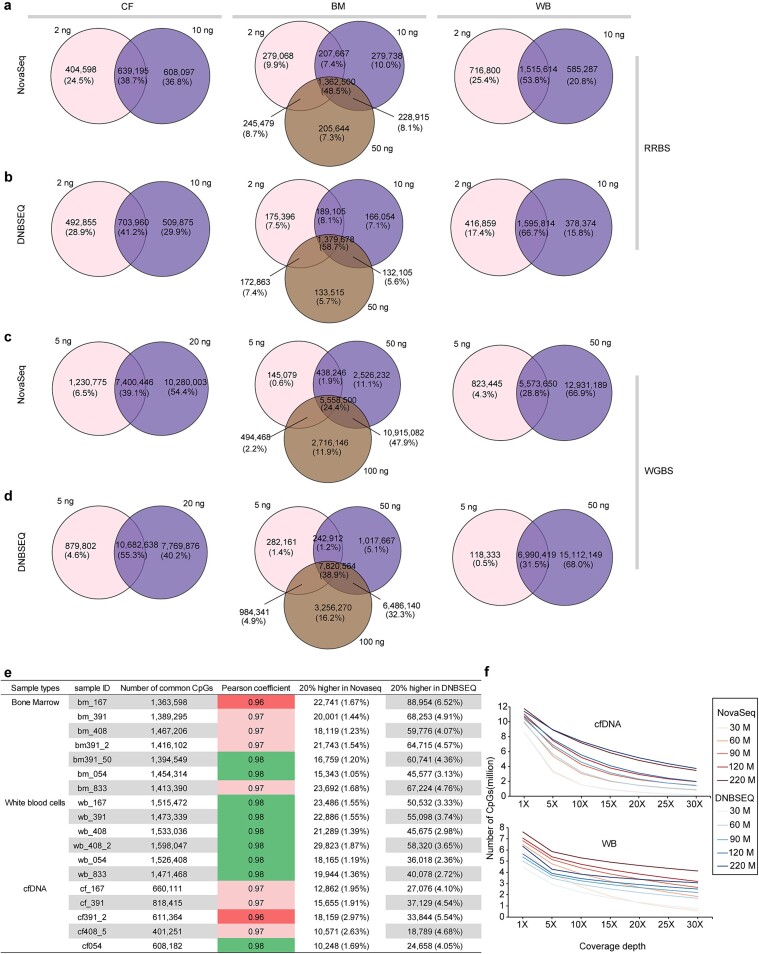
Comparison of intraplatform reproducibility and interplatform concordance for RRBS and WGBS. For RRBS, the overlap of CpG sites between different amounts of DNA inputs (2 and 10 ng for cfDNA; 2, 10, and 50 ng for genomic DNA of BM; 2 and 10 ng for genomic DNA of WBCs) was assessed for both (a) NovaSeq and (b) DNBSEQ. Venn diagrams illustrating platform-specific and platform-common CpGs between the platforms. The CpG methylation correlation profile between different DNA inputs for NovaSeq and DNBSEQ is shown in Supplementary Fig. S6. Similarly, for WGBS, the overlap of CpGs between different DNA inputs (5 and 20 ng for cfDNA; 5, 50, and 100 ng for BM genomic DNA; 5 and 50 ng for WB genomic DNA) was compared for (c) NovaSeq and (d) DNBSEQ, with the correlation of CpG methylation between different DNA inputs depicted in Supplementary Fig. S7. Interplatform concordance and differences for RRBS and WGBS are shown in (e) and Supplementary Table S1, detailing the number of common CpGs with methylation differences greater than 20% on both platforms. In addition, RRBS was performed on cfDNA and WB genomic DNA from two additional MDS patients with larger data volumes. (f) The number of observed CpGs at coverage depths ranging from 1× to 30× for one MDS patient, comparing different amounts of mapped reads for both sequencing platforms.

For RRBS, both the 2 and 10 ng cfDNA samples from the same individual yielded a similar number of CpGs on both platforms. The number of common CpGs for these DNA inputs was also comparable across the two sequencing platforms (Fig. 5a and b). Similarly, for the genomic DNA of BMMNCs and WBCs, the number of common CpGs for varied DNA inputs was consistent between the two platforms (Fig. 5a and b). These findings suggest that 2 ng of input may be sufficient, equivalent to 10 ng for cfDNA and 10 or 50 ng for genomic DNA samples (Fig. 5a and b). Additionally, for cfDNA, genomic DNA of BMMNCs and WBCs, the percentage of common CpGs among various DNA inputs was greater on the DNBSEQ platform than on the NovaSeq platform (cfDNA: 41.2% on DNBSEQ versus 38.7% on NovaSeq; genomic DNA of BMMNCs: 58.7% on DNBSEQ versus 48.5% on NovaSeq; genomic DNA of WBCs: 66.7% on DNBSEQ versus 53.8% on NovaSeq). We calculated the pairwise Pearson correlation coefficient (PCC) for common CpGs among various DNA inputs and found high correlations within either platform (Supplementary Fig. S6a–c).

#### Intraplatform reproducibility of whole-genome bisulfite sequencing

For WGBS, we found that 20 ng of cfDNA is preferable for library preparation because it yields significantly more CpGs than 5 ng for both sequencing platforms (Fig. 5c and d). Similarly, for genomic DNA, 50 ng is appropriate because it yields significantly more CpGs than 5 ng and is comparable to 100 ng (Fig. 5c and d). In addition, we observed that the ratio of common CpGs among different DNA inputs was greater on the DNBSEQ platform than on the NovaSeq platform. However, the pairwise PCC was greater on the NovaSeq platform than on the DNBSEQ platform for varied inputs of all samples (Supplementary Fig. S7a–c), indicating better intraplatform reproducibility for the NovaSeq platform than for the DNBSEQ platform when performing WGBS.

#### Interplatform reproducibility

To explore the interplatform reproducibility of RRBS datasets, we calculated the number of common CpGs and the pairwise PCC between 18 paired samples sequenced by both platforms. We found that the PCC was at least 0.96 between platforms, and the proportion of a 20% difference in the CpG methylation level between platforms was acceptable (Fig. 5e). For WGBS, the PCC was slightly lower (at least 0.8), and the proportion of a 20% difference in the CpG methylation level was also very low (Supplementary Table S1).

#### Sequencing depth

To better understand the relationship between sequencing depth and the number of CpGs identified, we conducted deep-depth RRBS on two additional individuals. It appears that both sequencing platforms yield similar numbers of CpGs at different sequencing depths for cfDNA. However, for genomic DNA, NovaSeq can obtain significantly more CpGs than DNBSEQ platforms for the same number of mapped reads (Fig. 5f). Overall, these results indicate that both platforms exhibit robust performance when performing RRBS for cfDNA.

### Detection of differentially methylated CpG sites in patients with myelodysplastic syndrome

**Figure 6:**
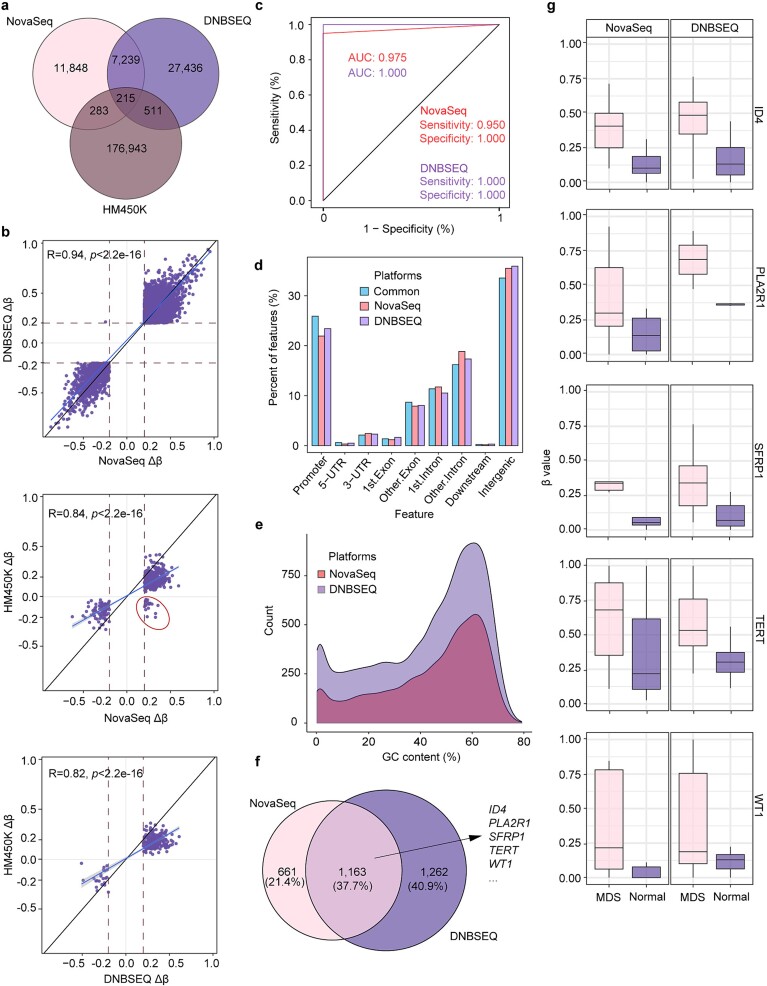
Comparison of DMP calling performance between the two sequencing platforms. For RRBS, DMPs were called using the MethylKit [46] package in R with a mean difference cutoff greater than 0.2. External HM450k data from a specific cohort were used as a reference to evaluate the accuracy of both sequencing platforms. (a) Venn diagram illustrating the number of overlapping DMPs between the three platforms. (b) Scatter plot showing the distribution of methylation differences for DMPs between any two platforms. (c) The sensitivity, specificity, and AUC were calculated for both platforms using the HM450k array data as a reference. (d) The distribution of DMPs across different genomic features was analyzed for platform-specific and common DMPs. (e) The distribution of DMPs based on different GC contents was plotted to investigate potential bias related to GC content. (f) DMPs identified by one or both platforms were mapped to gene promoters, and their overlapping genes were visualized using a Venn diagram. (g) A box plot showing the methylation levels of gene promoters associated with MDS, demonstrating the ability of both platforms to detect aberrant methylation in MDS patients.

While previous sections have focused on comparing CpG coverage and methylation levels, the ability of the two platforms to identify DMPs remains unclear. We then quantitatively compared the performance of DMP calling between the two sequencing platforms using RRBS datasets of BM samples from both MDS patients and healthy donors. In addition, we introduced external data generated using the Illumina HumanMethylation450 BeadChip (HM450k) as a reference, as HM450k is widely utilized for The Cancer Genome Atlas (TCGA) and various other large-scale sequencing projects [66, 67]. Hence, we utilized an MDS cohort from the TCGA database, in which DMPs between MDS patients and healthy controls were detected using the HM450k chip. These DMPs were then compared with those identified in this study through the use of the two sequencing platforms. Notably, the DNBSEQ platform detected more DMPs than NovaSeq due to a greater proportion of overlapping CpGs (Fig. 6a). Specifically, we detected 7454 DMPs in both sequencing platforms, with a PCC of 0.94, and almost all the DMPs exhibited the same direction for hyper or hypomethylated sites (Fig. 6b). When the sequencing data were compared to the reference HM450 array data, it was observed that all DMPs exhibited the same direction of hyper or hypomethylated sites on the DNBSEQ platforms, while for NovaSeq, a small fraction of DMPs had opposite directions compared to the HM450 array data. The area under the curve (AUC) for DNBSEQ and NovaSeq was 1 and 0.975, respectively, as shown in Fig. 6c. Despite the obvious differences in the number of DMPs detected by the two sequencing platforms, the genomic feature distributions of the DMPs were similar (Fig. 6d). To assess any potential bias caused by GC content, we assigned DMPs to different GC contents using non-overlapping 1000 bp windows across the whole genome and found that both platforms had similar frequency distributions of DMPs across different GC contents (Fig. 6e).

Additionally, these DMPs were mapped to the promoter regions of 1824 and 2425 genes for NovaSeq and DNBSEQ, respectively, and 37.7% of these genes were found to be differentially methylated on both platforms. Within the overlapping gene list, we found that some well-studied aberrantly methylated genes in MDS were present (Fig. 6f) [68–74]. Furthermore, we plotted the methylation status of promoters for five genes based on the beta value and observed that these promoters were hypermethylated in MDS patients compared to healthy controls on both platforms (Fig. 6g), which is consistent with previous studies [68–74]. Moreover, we performed Gene Ontology enrichment analysis on two sets of gene lists from each platform, and the results showed that 12 of the top 20 enriched gene pathways were the same (Supplementary Fig. S8a and b). These findings indicate that both platforms are capable of detecting aberrantly methylated genes.

## Discussion

In this study, we systematically evaluated the performance of two widely used large-scale NGS platforms (Supplementary Table S2), the Illumina NovaSeq 6000 and the MGI Tech DNBSEQ-T7, with a particular focus on their effectiveness in performing two commonly used bisulfite sequencing techniques: WGBS and RRBS [8, 9, 27]. Unlike previous platform comparison studies focused on WGS, WES, or RNA-seq, which do not involve bisulfite conversion in library preparation procedures [14, 15, 21, 22], WGBS and RRBS based on NGS platforms face greater challenges due to reduced base complexity, GC bias and PCR bias [62]. Therefore, we designed a comprehensive study to investigate the potential sources of bias introduced by these sequencing platforms in the context of WGBS and RRBS. We found better base quality for DNBSEQ than for NovaSeq, which is consistent with previous studies [75, 76]. However, the reported base quality scores may not reflect the true scores [51], so we used the BQSR to evaluate the gap between the true and reported base quality scores. The BQSR procedure for bisulfite sequencing reads is somewhat different from that for nonbisulfite sequencing reads, as it cannot tell whether a base is truly bisulfite converted or just an SNP. Therefore, we carefully excluded sites that could cause confusion. Due to the use of a streamlined quality scoring method with real-time analysis 3 (RTA3) software adopted by NovaSeq [77], it was not possible to obtain the full raw base quality scores for NovaSeq (instead, the scores were binned to 2, 11, 25, and 37 in the FASTQ file of this paper). However, from the results available, we can still see that both platforms overestimate the base quality scores. The differences in the trimming, mapping and duplication ratio metrics between the two sequencing platforms were significant, and similar results have been found in other studies [78, 79]. In addition, we observed a bias toward smaller insert sizes in the DNBSEQ datasets for RRBS and WGBS (Fig. 2i and j), which is in agreement with a previous study [78]. We speculate that the fragment size bias may be attributed to the DNB sequencing process, which relies on RCA to generate sequencing libraries. RCA has been reported to favor smaller DNA fragments [80]. Not surprisingly, the mapping ratio of RRBS libraries from the NovaSeq platform is lower than that of the DNBSEQ platform (Fig. 2k), as the latter generates more high-quality bases, resulting in more sequencing reads that align to the reference genome. Likewise, the relatively lower proportion of high-quality bases from the NovaSeq platform may contribute to the higher error rates in the WGBS libraries (Fig. 2n).

The differences in sequencing performance prompted us to further explore the characteristics of CpG coverage and methylation for the two platforms, which are critical for identifying DMPs or differentially methylated regions. To rule out the influence of sequencing depth, we downsampled the data from each library to the same number of mapped reads. The RRBS results revealed that genomic DNA samples sequenced with DNBSEQ covered a smaller fraction of the genome than those sequenced with NovaSeq, leading to a reduced number of CpG sites identified by DNBSEQ. The observation is consistent with previous reports [14, 78]. However, this difference was less pronounced for RRBS libraries generated using cfDNA, the majority of which is less than 200 bp [81]. These results may indicate that both platforms are robust in recovering CpGs from cfDNA. In contrast, some CpGs are lost during DNBSEQ sequencing of RRBS libraries generated from genomic DNA with a broad size distribution, likely due to RCA-associated loss of larger library DNA fragments [27] (Supplementary Fig. S9a–c). Not only did the two platforms differ in the number of CpGs recovered, but a genomic region bias was also found, with a larger proportion of CpGs in DNBSEQ occurring more frequently in intergenic regions and less frequently in promoter regions.

For critical functional regions such as promoters, CGIs and enhancers, the mean coverage was greater in NovaSeq. Given the significant bias in coverage breadth and depth, we suspected that methylation levels might also be affected. It turned out that the platform-specific covered CpGs in DNBSEQ mainly consisted of highly methylated sites, indicating a bias to highly methylated CpGs for the platform. However, for the common CpG sites of both platforms, the proportions of hypermethylated and hypomethylated sites were comparable. Combining the small differences in mean methylation across all three types of functional regions and the high correlation coefficients for pairwise samples between the two platforms, we believe that both platforms can accurately estimate CpG methylation in RRBS. For WGBS, we discovered more uneven coverage across different CpG contents and dinucleotides in the DNBSEQ platform, which also exhibited a bias toward highly methylated CpGs compared to NovaSeq. To understand how the coverage bias of the platforms affects the methylation level estimation, we compared the mean coverage between GC-rich regions (the top 1000 CGIs by GC content) and GC-poor regions (the bottom 1000 CGIs) and found that GC-rich regions had higher mean coverage and were more likely to have low methylation. In contrast, GC-poor regions had less variation in mean coverage and tended to be highly methylated. This explains why DNBSEQ shows a coverage bias toward highly methylated regions. Interplatform comparisons of the methylation levels of shared CpGs revealed Pearson coefficients ranging from 0.8 to 0.95. However, DNBSEQ displayed lower intraplatform Pearson coefficients than NovaSeq, suggesting a lower efficacy of DNBSEQ in WGBS applications.

To assess the accuracy of RRBS in detecting DMPs, we used HM450K array data from a large cohort of MDS patients from the TCGA project [82]. Our analysis revealed a high correlation between the DMPs identified by both platforms and the reference data, with DNBSEQ showing slightly greater accuracy. These results suggest that researchers should choose between these two sequencing platforms based on their specific need to obtain reliable methylation measurements.

Several strategies can be employed to improve reproducibility within a single platform and concordance between different platforms for RRBS and WGBS. First, standardization of library preparation and sequencing protocols is critical to minimize variability [27, 83]. Using consistent bioinformatics pipelines for alignment and methylation measurement ensures comparability [84]. In addition, generating reference libraries for calibration and performing technical replicates can help identify and correct inconsistencies. Finally, developing cross-platform normalization techniques and data integration frameworks can mitigate platform-specific biases and improve overall data consistency and reliability [85].

## Conclusions

Our results revealed distinct characteristics of two commercially available large-scale sequencing platforms for profiling genome-wide methylation using two widely accepted bisulfite sequencing technologies. Given the substantial differences in sequencing principles between the Illumina and MGI Tech platforms, the bias caused by sequencing platforms should be carefully considered when employing different sample types, different DNA inputs, WGBS or RRBS on any sequencing platform. We anticipate that our findings will benefit both sequencing instrument manufacturers and researchers in the epigenetics community by improving sequencing performance and accurately profiling DNA methylation changes.

**Key Points**

- We performed systematic comparisons between two sequencing platforms for genome-wide bisulfite sequencing.
- Different sources of samples and DNA inputs showed distinct characteristics for RRBS and WGBS.
- Our findings will benefit both sequencing instrument manufacturers and researchers in the epigenetics community by improving sequencing performance and accurately profiling DNA methylation changes.

## Supplementary Material

Revised_Supplemental_File_bbae440

## Data Availability

The RRBS and WGBS datasets generated in this study were deposited in the Genome Sequence Archive (GSA) under accession number HRA006653 under controlled access. Access can be requested through Fan Zhang (fzhang@cmpt.ac.cn) and will be made available for noncommercial use for a minimum of 5 years.
