# Supplementary material for: Beyond the base pairs: comparative genome-wide DNA methylation profiling across sequencing technologies
Source: Brief Bioinform. 2024 Sep 10;25(5):bbae440. doi: 10.1093/bib/bbae440 (PMC11387064; doi:10.1093/bib/bbae440)
Supplement: Revised_Supplemental_File_bbae440 [file revised_supplemental_file_bbae440.pdf]

Supplementary Figures:

Figure S1. Base quality recalibration for WGBS data from NovaSeq and DNBSEQ. (a) Scatter plots show the empirical quality score and the reported quality score before and after recalibration for both platforms. (b) Bar graphs illustrate the distribution of quality scores. (c) and (d) exhibit the quality score accuracy and mean quality score for each read cycle, respectively. (e) and (f) show the mean quality score accuracy and mean quality score in different dinucleotide contexts, separately.

Figure S2. Common CpGs among RRBS libraries from three MDS patients and two healthy donors sequenced on NovaSeq and DNBSEQ platforms.

Figure S3. Comparison of average methylation levels in genomic regions using NovaSeq and DNBSEQ RRBS datasets. Graphs show average methylation in promoters (a), CpG islands (b), and enhancers (c), with detailed analysis described in the Methods section.

Figure S4. Genome coverage at different depths for two types of WGBS libraries. The figure displays data for eight libraries of each type.

Figure S5: Comparison of normalized coverage and average methylation levels from two types of WGBS data. (a) The normalized coverage for the top 1,000 CpG islands (CGIs) with the highest GC content and for the bottom 1,000 CGIs with the lowest GC content. (b) The average methylation levels for promoters. (c) The average methylation levels for enhancers. (d) The average methylation levels for the top 1,000 CGIs with the highest GC content and for the bottom 1,000 CGIs with the lowest GC content.

Figure S6. Correlation of CpG methylation levels between RRBS libraries with different DNA inputs. Correlation coefficients were calculated and visualized using the GetCorrelation function in methylKit.

Figure S7. Correlation of CpG methylation levels between WGBS libraries with different DNA inputs. Correlation coefficients were calculated and visualized using the GetCorrelation function in methylKit.

Figure S8. Top 20 pathways with aberrantly methylated genes in MDS patients identified by GO enrichment analysis of RRBS data. Pathways enriched across platforms are indicated by identical numerical labels for easy cross-referencing.

Figure S9. Distribution of insert size for NovaSeq and DNBSEQ RRBS datasets. The number of mapped reads in all RRBS libraries are downsampled to the same.

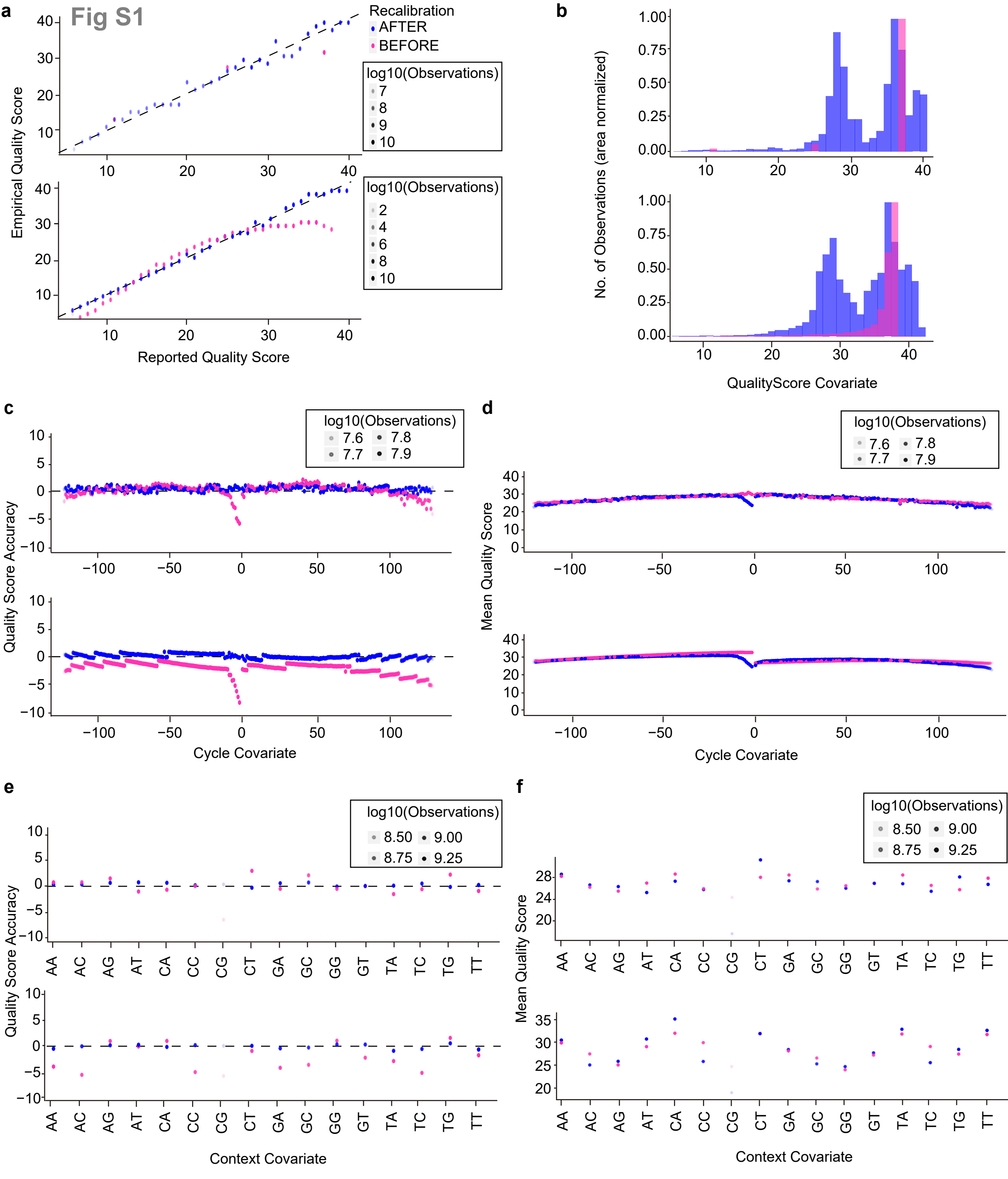
Fig S1
a
Recalibration
AFTER
BEFORE
log10(Observations)
Empirical Quality Score
Reported Quality Score
b
No. of Observations (area normalized)
QualityScore Covariate
c
Quality Score Accuracy
Cycle Covariate
d
Mean Quality Score
Cycle Covariate
e
Quality Score Accuracy
Context Covariate
f
Mean Quality Score
Context Covariate

Fig S2

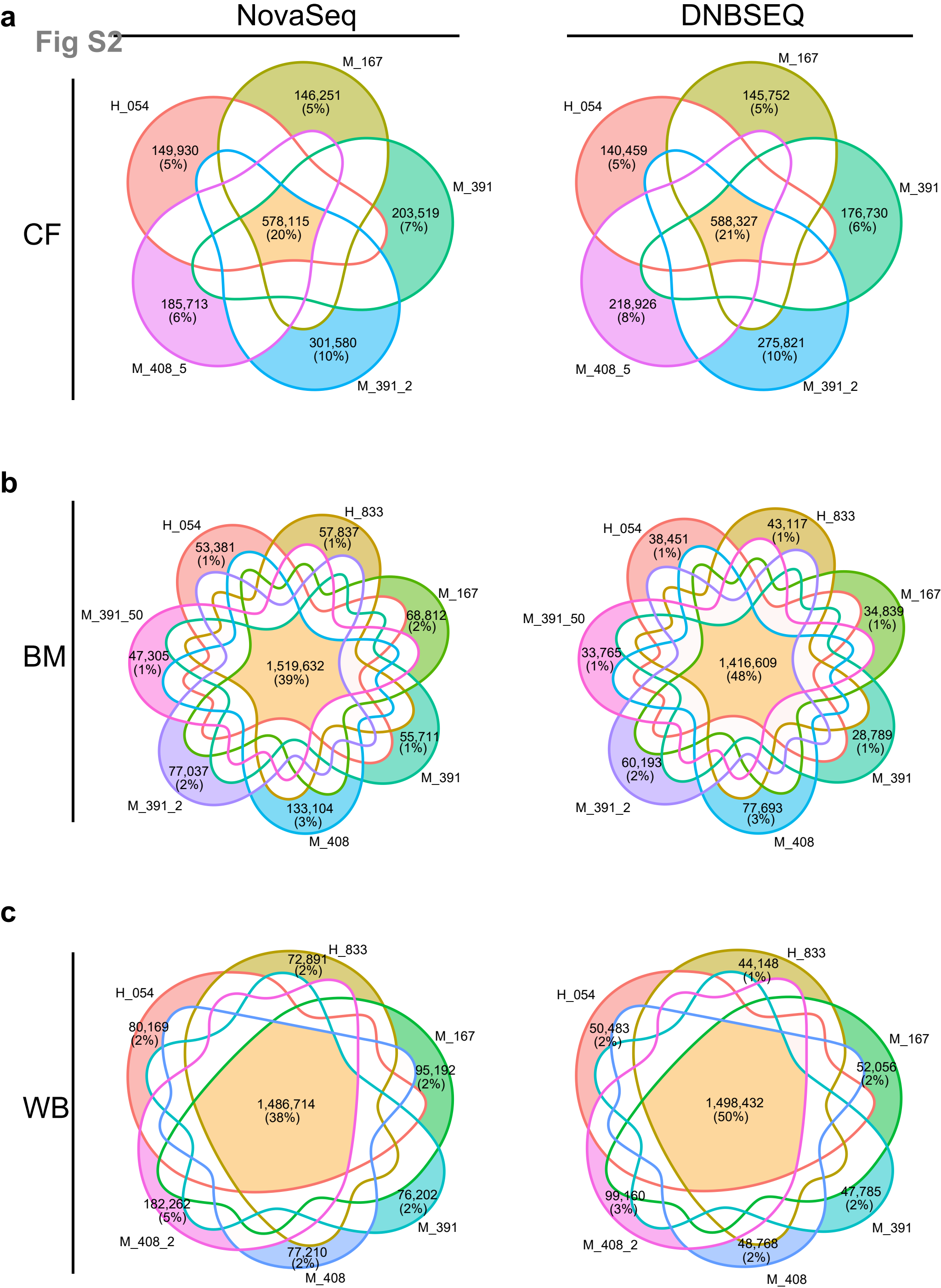
a
NovaSeq
DNBSEQ
CF
H_054
M_167
M_391
M_391_2
M_408_5
149,930 (5%)
146,251 (5%)
203,519 (7%)
301,580 (10%)
185,713 (6%)
578,115 (20%)
140,459 (5%)
145,752 (5%)
176,730 (6%)
275,821 (10%)
218,926 (8%)
588,327 (21%)
b
BM
H_054
H_833
M_167
M_391
M_408
M_391_2
M_391_50
53,381 (1%)
57,837 (1%)
68,812 (2%)
55,711 (1%)
133,104 (3%)
77,037 (2%)
47,305 (1%)
1,519,632 (39%)
38,451 (1%)
43,117 (1%)
34,839 (1%)
28,789 (1%)
77,693 (3%)
60,193 (2%)
33,765 (1%)
1,416,609 (48%)
c
WB
H_054
H_833
M_167
M_391
M_408
M_408_2
80,169 (2%)
72,891 (2%)
95,192 (2%)
76,202 (2%)
77,210 (2%)
182,262 (5%)
1,486,714 (38%)
50,483 (2%)
44,148 (1%)
52,056 (2%)
47,785 (2%)
48,768 (2%)
99,160 (3%)
1,498,432 (50%)

Fig S3

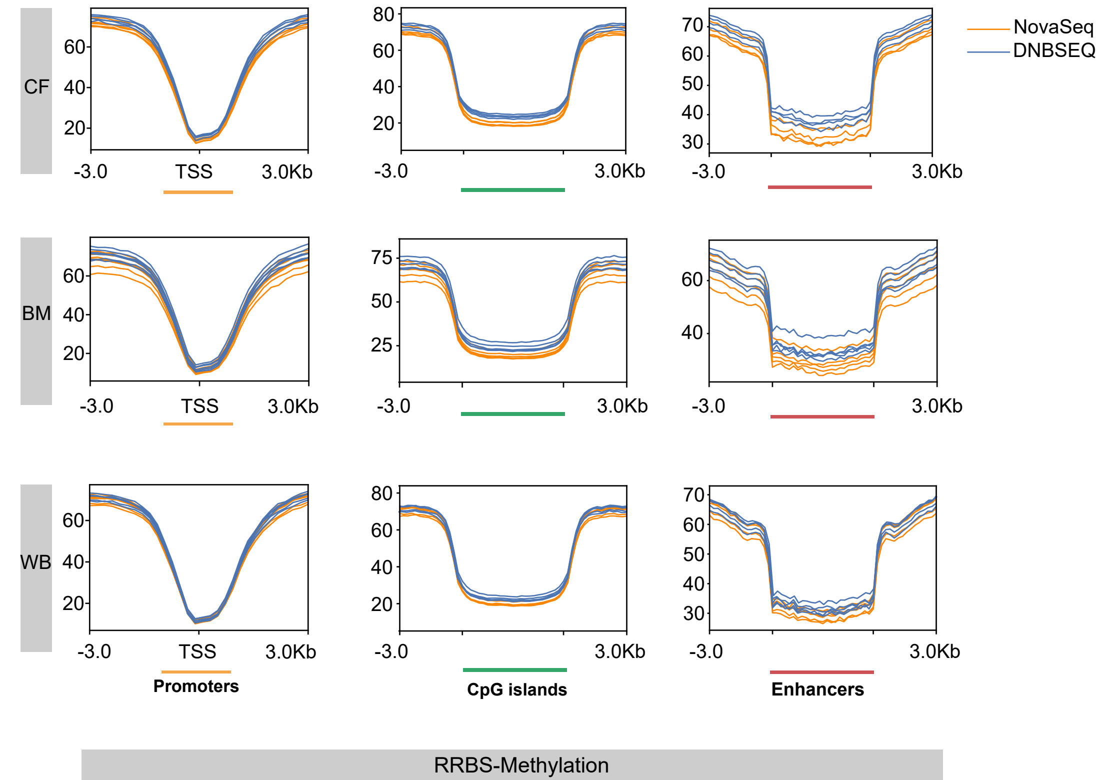

a
b
c
CF
BM
WB
NovaSeq
DNBSEQ
-3.0
TSS
3.0Kb
Promoters
CpG islands
Enhancers
RRBS-Methylation

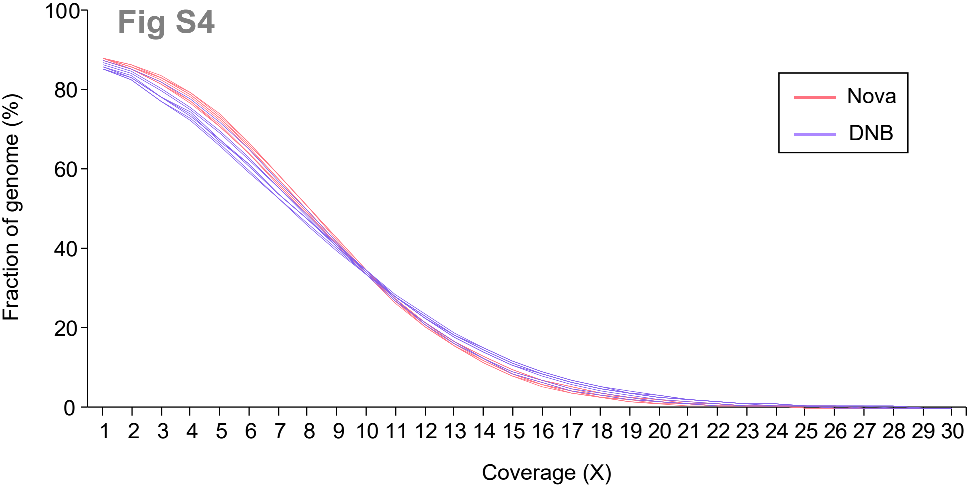
Fig S4
Fraction of genome (%)
Coverage (X)
Nova
DNB
0
20
40
60
80
100
1 2 3 4 5 6 7 8 9 10 11 12 13 14 15 16 17 18 19 20 21 22 23 24 25 26 27 28 29 30

Fig S5

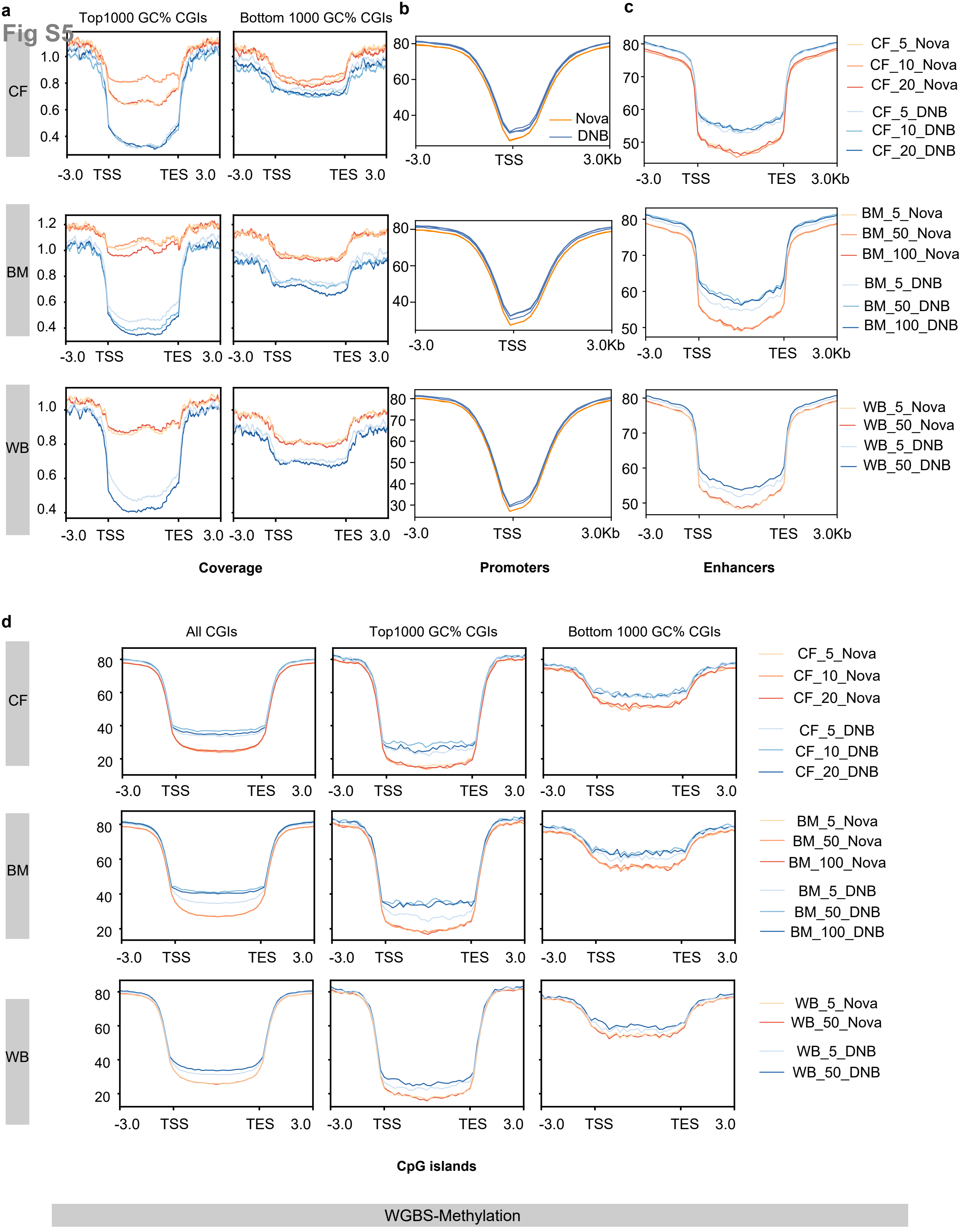
a
Top1000 GC% CGIs
Bottom 1000 GC% CGIs
CF
BM
WB
1.2
1.0
0.8
0.6
0.4
-3.0 TSS TES 3.0
Coverage
b
80
60
40
30
Nova
DNB
-3.0 TSS 3.0Kb
Promoters
c
80
70
60
50
-3.0 TSS TES 3.0Kb
CF_5_Nova
CF_10_Nova
CF_20_Nova
CF_5_DNB
CF_10_DNB
CF_20_DNB
BM_5_Nova
BM_50_Nova
BM_100_Nova
BM_5_DNB
BM_50_DNB
BM_100_DNB
WB_5_Nova
WB_50_Nova
WB_5_DNB
WB_50_DNB
Enhancers
d
All CGIs
Top1000 GC% CGIs
Bottom 1000 GC% CGIs
80
60
40
20
-3.0 TSS TES 3.0
CpG islands
WGBS-Methylation

Fig S6

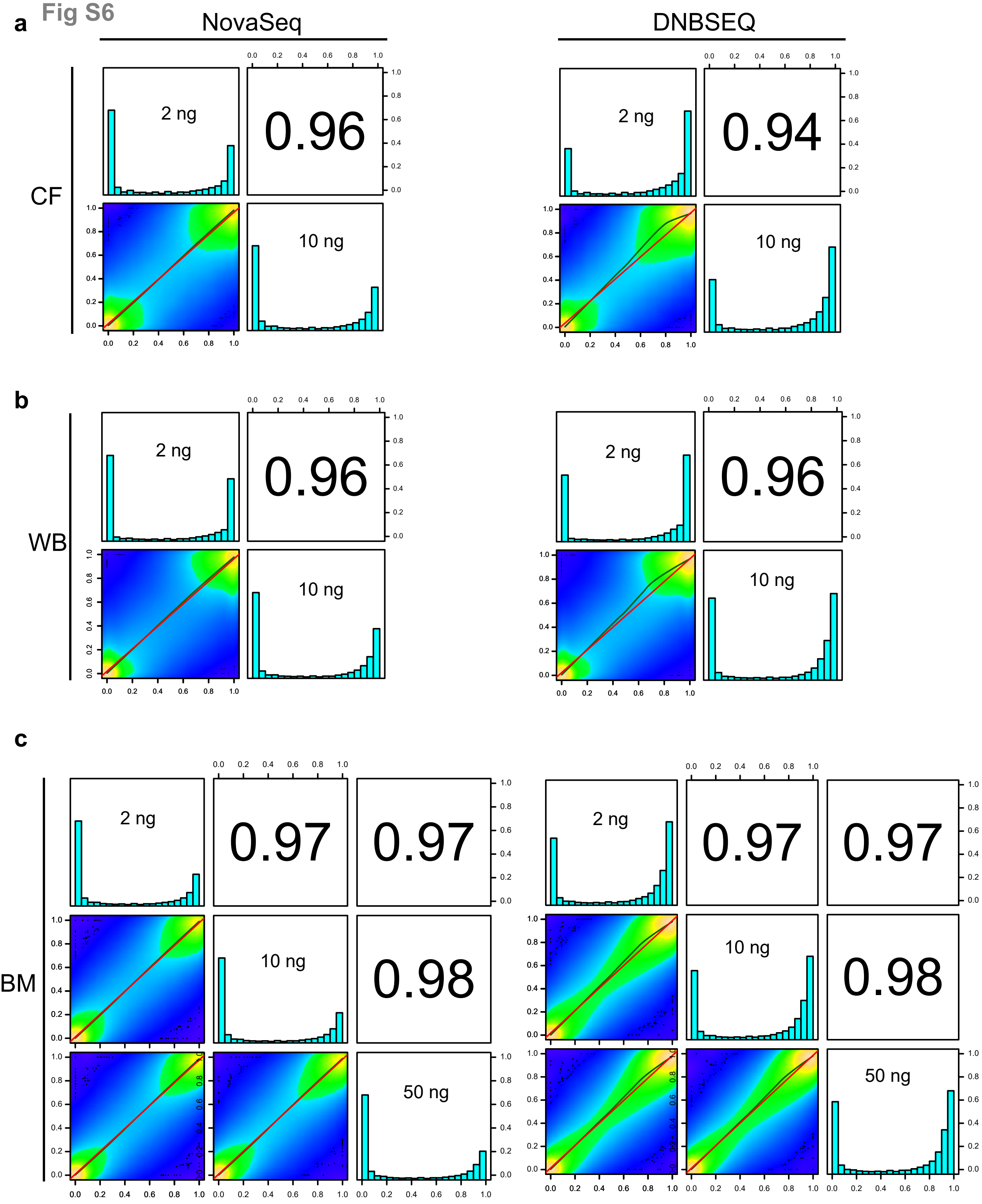
a
NovaSeq
DNBSEQ
CF
2 ng
0.96
10 ng
2 ng
0.94
10 ng
b
WB
2 ng
0.96
10 ng
2 ng
0.96
10 ng
c
BM
2 ng
0.97
0.97
10 ng
0.98
50 ng
2 ng
0.97
0.97
10 ng
0.98
50 ng

Fig S7

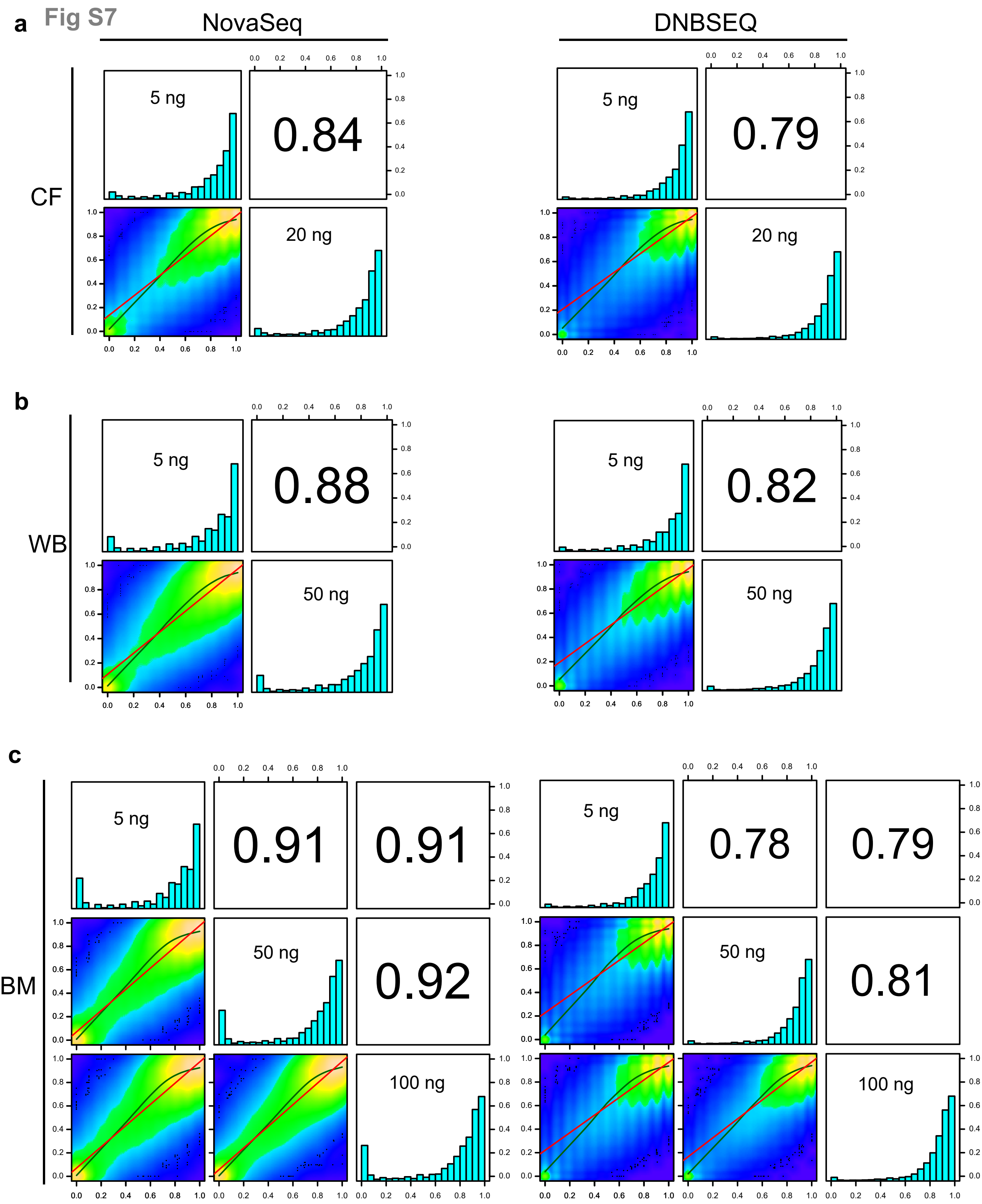

a
NovaSeq
DNBSEQ
CF
5 ng
0.84
20 ng
5 ng
0.79
20 ng
b
WB
5 ng
0.88
50 ng
5 ng
0.82
50 ng
c
BM
5 ng
0.91
0.91
50 ng
0.92
100 ng
5 ng
0.78
0.79
50 ng
0.81
100 ng

Fig S8

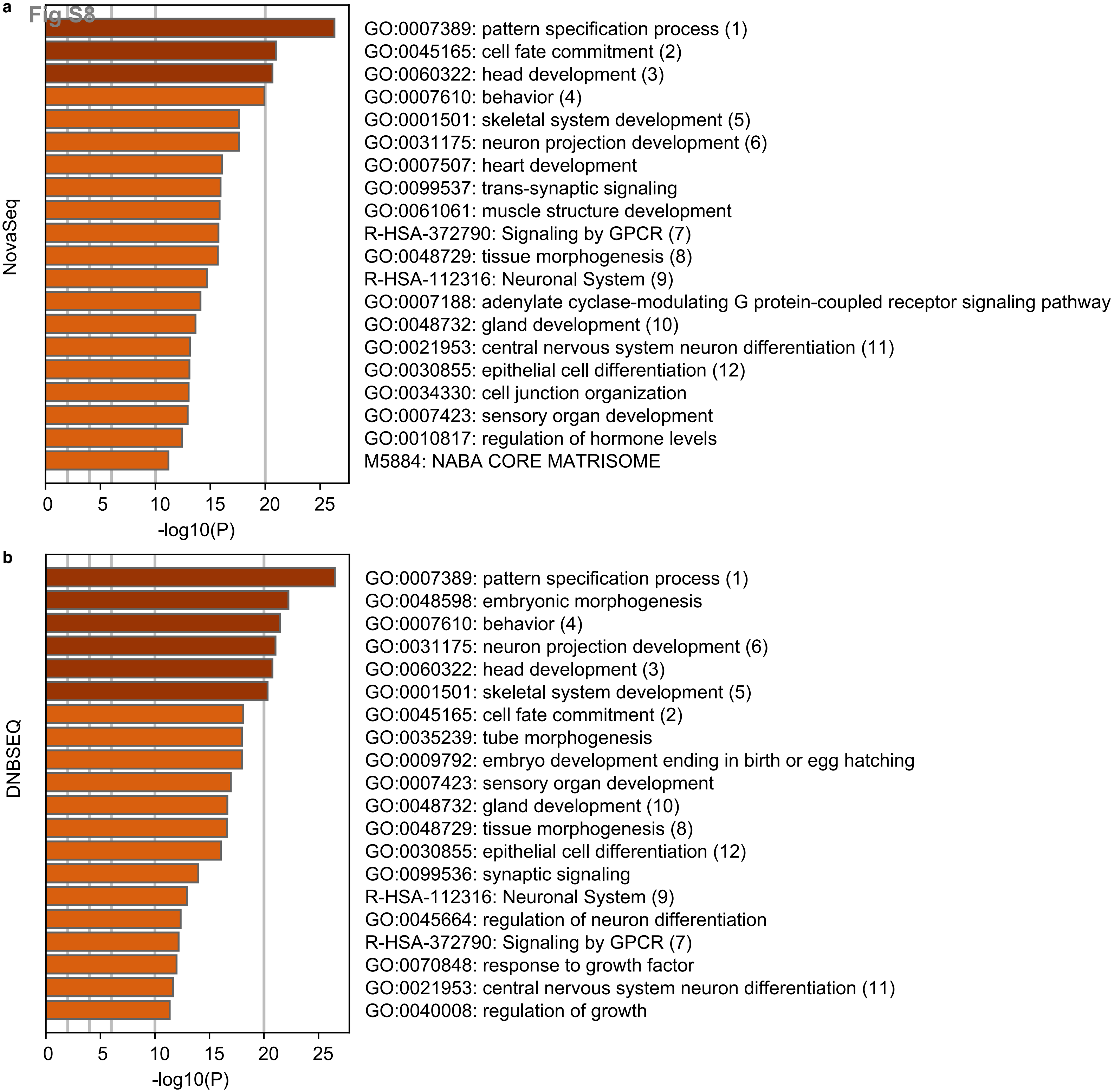
a
NovaSeq
GO:0007389: pattern specification process (1)
GO:0045165: cell fate commitment (2)
GO:0060322: head development (3)
GO:0007610: behavior (4)
GO:0001501: skeletal system development (5)
GO:0031175: neuron projection development (6)
GO:0007507: heart development
GO:0099537: trans-synaptic signaling
GO:0061061: muscle structure development
R-HSA-372790: Signaling by GPCR (7)
GO:0048729: tissue morphogenesis (8)
R-HSA-112316: Neuronal System (9)
GO:0007188: adenylate cyclase-modulating G protein-coupled receptor signaling pathway
GO:0048732: gland development (10)
GO:0021953: central nervous system neuron differentiation (11)
GO:0030855: epithelial cell differentiation (12)
GO:0034330: cell junction organization
GO:0007423: sensory organ development
GO:0010817: regulation of hormone levels
M5884: NABA CORE MATRISOME
0
5
10
15
20
25
-log10(P)
b
DNBSEQ
GO:0007389: pattern specification process (1)
GO:0048598: embryonic morphogenesis
GO:0007610: behavior (4)
GO:0031175: neuron projection development (6)
GO:0060322: head development (3)
GO:0001501: skeletal system development (5)
GO:0045165: cell fate commitment (2)
GO:0035239: tube morphogenesis
GO:0009792: embryo development ending in birth or egg hatching
GO:0007423: sensory organ development
GO:0048732: gland development (10)
GO:0048729: tissue morphogenesis (8)
GO:0030855: epithelial cell differentiation (12)
GO:0099536: synaptic signaling
R-HSA-112316: Neuronal System (9)
GO:0045664: regulation of neuron differentiation
R-HSA-372790: Signaling by GPCR (7)
GO:0070848: response to growth factor
GO:0021953: central nervous system neuron differentiation (11)
GO:0040008: regulation of growth
0
5
10
15
20
25
-log10(P)

Fig S9

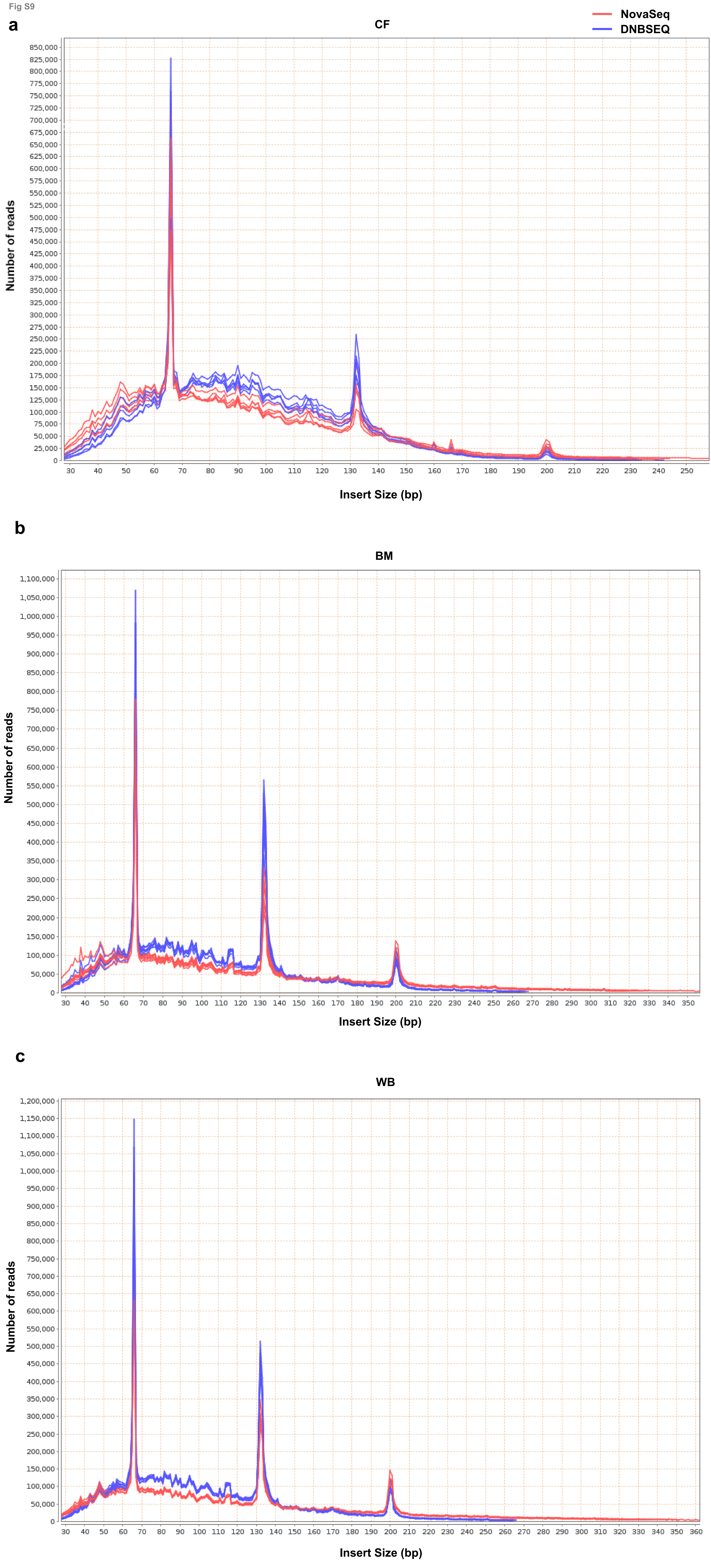
a
CF
NovaSeq
DNBSEQ
Number of reads
Insert Size (bp)
b
BM
Number of reads
Insert Size (bp)
c
WB
Number of reads
Insert Size (bp)

Table S1. Inter-platform comparison of CpG methylation correlation between NovaSeq and DNBSEQ.

Sample type	Input (ng)	Common CpGs	Pearson coefficient	20% higher in NovaSeq	20% higher in DNBSEQ
BM gDNA	100	6,751,548	0.83	2 (0.00%)	19 (0.00%)
	50	4,807,858	0.81	147,695 (3.07%)	269,711 (5.61%)
	5	371,642	0.89	6,630 (1.78%)	10,847 (2.92%)
WBC gDNA	50	7,875,330	0.88	210,387 (2.67%)	345,170 (4.38%)
	5	531,521	0.95	3,162 (0.59%)	4,345 (0.82%)
cfDNA	20	6,969,770	0.83	203,684 (2.92%)	325,915 (4.68%)
	5	1,540,106	0.85	40,580 (2.63%)	63,631 (4.13%)
	10	2,224,041	0.8	75,601 (3.40%)	126,862 (5.70%)

Table S2. Summary of the performance comparison between NovaSeq 6000 and DNBSEQ-T7.

**Metrics**	**NovaSeq**				**DNBSEQ**			
	RRBS		WGBS		RRBS		WGBS	
	gDNA	cfDNA	gDNA	cfDNA	gDNA	cfDNA	gDNA	cfDNA
≥Q20	91	90	96	95	97	97	97	97
≥Q30	83	82	90	90	91	92	91	91
Trimming (%)	19	22	3	6	5	5	0	0
Duplication (%)	-	-	30	23	-	-	21	10
Mapping ratio (%)	66	65	70	73	72	75	68	69
Error mapping ratio (%)	0.58	0.56	0.19	0.18	0.66	0.54	0.18	0.18
Insert size (bp)	214	163	186	159	165	140	164	142
MethylC level (%)	50	57	78	80	62	69	83	84
Number of CpGs (≥10X)	2,708,696	1,583,931	3,215,637	1,955,192	2,228,953	1,525,034	2,559,853	1,944,407
Intra-platform reproducibility	0.97	0.96	0.91	0.84	0.97	0.94	0.80	0.79
